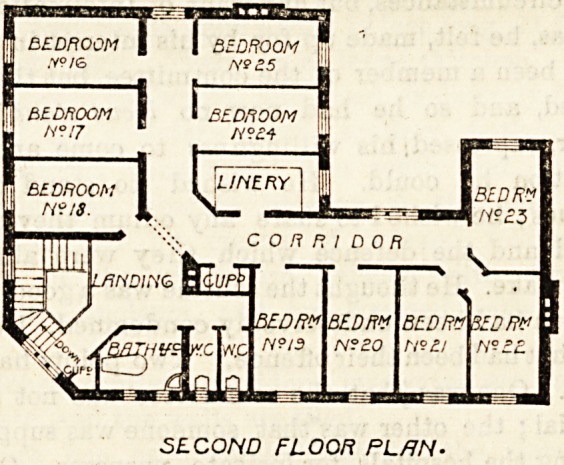# New Home for Nurses at the Cancer Hospital, Fulham Road, S.w.

**Published:** 1904-11-19

**Authors:** 


### NEW HOME FOR NURSES AT THE CANCER HOSPITAL, FULHAM ROAD, S.W.

For some time the night nurses of this hospital have been provided for in a separate building in a quiet part of the grounds, and now a new home for the day-nurses is being built. The ground plan of this block shows that the

GROUND FLOOR PLAN.

entrance hall is at the north-east angle, and here are also the staircases and lavatory. A rectangular corridor gives access to the various rooms, there being on this floor two good sitting-rooms, a pantry and five bedrooms. There is also a verandah facing almost due south. The first floor contains 10 bedrooms, two bath-rooms and closets, and there is a balcony over the ground floor verandah. The second floor is a replica of the first floor, except that one of the bath-rooms is made into a linen-room, and that the balcony is not carried up. There is, therefore, a total of 25 bedrooms on the three floors, the intention being evidently to give each nurse a room to herself. There is no dining-room in the block, because it is not intended that the nurses shall take their meals therein. Considering the ground space occupied by the block, the accommodation is very good. The corridors are well lighted and ventilated, and all the arrangements are satisfactory. We should, however, have preferred to see the closets projecting or even separated from the main building so as to obtain cross-ventilation by a short corridor. The committee of the hospital deserve credit for providing such a comfortable and compact annexe for their nursing staff, and it is certain that it will be

FIRST FLOOR PLAN.

SECOND FLOOR PLAN.

appreciated and that the better nurses are taken care of the better they will perform their duties. The architect is Mr. Alexander Graham, of Carlton Chambers, Regent Street, and the contractors are Messrs. Higgs and Hill. The cost will be about £5,000.

## Figures and Tables

**Figure f1:**
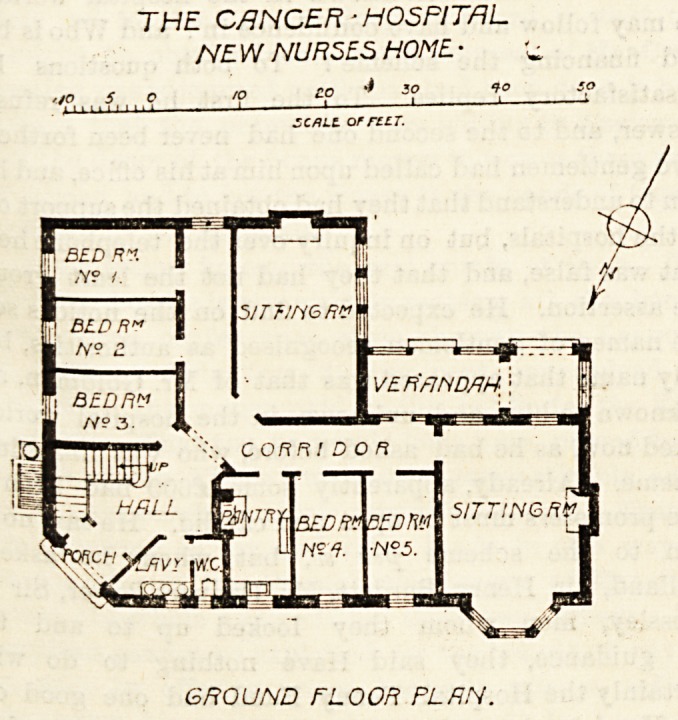


**Figure f2:**
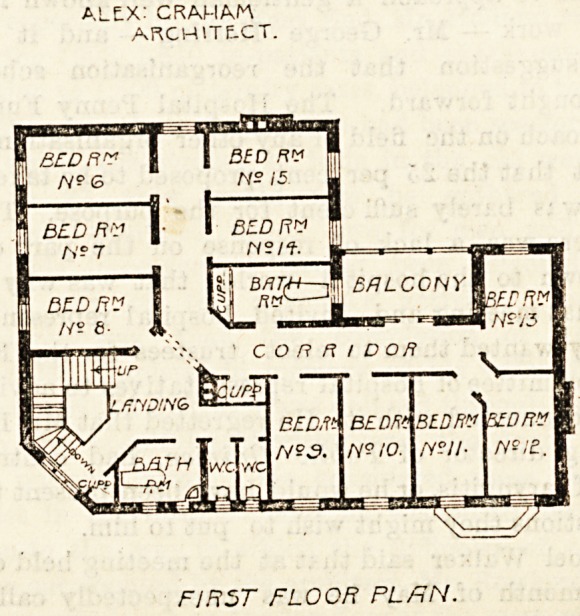


**Figure f3:**